# Genetic and pharmacologic inhibition of Tpl2 kinase is protective in a mouse model of ventilator-induced lung injury

**DOI:** 10.1186/2197-425X-2-15

**Published:** 2014-05-09

**Authors:** Evangelos Kaniaris, Katerina Vaporidi, Eleni Vergadi, Emmanuel E Theodorakis, Eumorfia Kondili, Eleni Lagoudaki, Christos Tsatsanis, Dimitris Georgopoulos

**Affiliations:** Department of Intensive Care Medicine, Experimental Intensive Care Medicine Laboratory, University of Crete, School of Medicine, Heraklio, Crete, 71003 Greece; Department of Pathology, University of Crete, School of Medicine, Heraklio, Crete, 70013 Greece; Department of Clinical Chemistry, University of Crete, School of Medicine, Heraklio, Crete, 71003 Greece

**Keywords:** Tumor progression locus 2, Tpl2 inhibitor, Extracellular signal-regulated kinase, Lung injury, Mechanical stress, Mechanical ventilation

## Abstract

**Background:**

Mechanical stress induced by injurious ventilation leads to pro-inflammatory cytokine production and lung injury. The extracellular-signal-regulated-kinase, ERK1/2, participates in the signaling pathways activated upon mechanical stress in the lungs to promote the inflammatory response. Tumor progression locus 2 (Tpl2) is a MAP3kinase that activates ERK1/2 upon cytokine or TLR signaling, to induce pro-inflammatory cytokine production. The role of Tpl2 in lung inflammation, and specifically in the one caused by mechanical stress has not been investigated. The aim of the study was to examine if genetic or pharmacologic inhibition of Tpl2 could ameliorate ventilator-induced lung injury.

**Methods:**

Adult male wild-type and Tpl2-deficient mice were ventilated with normal or high tidal volume for 4 h. Additional wild-type mice were treated with a Tpl2 inhibitor either before or 30 min after initiation of high tidal ventilation. Non-ventilated mice of both genotypes served as controls. The development of lung injury was evaluated by measuring lung mechanics, arterial blood gases, concentrations of proteins, IL-6, and MIP-2 in bronchoalveolar lavage fluid (BALF) and by lung histology. Data were compared by Kruskal-Wallis non-parametric test and significance was defined as *p* < 0.05.

**Results:**

Mechanical ventilation with normal tidal volume induced a mild increase of IL-6 in BALF in both strains. High tidal volume ventilation induced lung injury in wild-type mice, characterized by decreased lung compliance, increased concentrations of proteins, IL-6 and MIP-2 in BALF, and inflammatory cell infiltration on histology. All indices of lung injury were ameliorated in Tpl2-deficient mice. Wild-type mice treated with the Tpl2 inhibitor, either prior of after the initiation of high tidal volume ventilation were protected from the development of lung injury, as indicated by preserved lung compliance and lower BALF concentrations of proteins and IL-6, than similarly ventilated, untreated wild-type mice.

**Conclusions:**

Genetic and pharmacologic inhibition of Tpl2 is protective in a mouse model of ventilator-induced lung injury, ameliorating both high-permeability pulmonary edema and lung inflammation.

## Background

Ventilator-induced lung injury (VILI) is a well-recognized complication of mechanical ventilation. The mechanical stress caused by a relatively high tidal volume, applied on an injured lung causes an abnormally high distortion (strain) of lung cells. The cellular responses to increased stress and strain result in alveolar barrier disruption and activation of inflammation, therefore inducing or exacerbating acute lung injury (ALI).

Several cell types mediate the effects of strain induced by mechanical ventilation. Increased strain, either *in vivo* or *in vitro*, impairs barrier properties of alveolar epithelial and endothelial cells [1–3]. Moreover, both alveolar epithelial and endothelial cells have been shown to produce pro-inflammatory cytokines when subjected to deformation [4, 5]. Alveolar macrophages are also activated by cyclic stretch [6], and have been shown to mediate not only inflammation, but also barrier dysfunction [6–8].

The extracellular signal-regulated kinase 1/2 (ERK1/2) is a member of the mitogen-activated serine/threonine kinase (MAPK) family, which also includes the kinases p38 and C-Jun N-terminal Kinase (JNK). MAPK are highly conserved enzymes regulating a vast array of cellular functions, including cell survival, proliferation, and differentiation, as well as inflammation and stress responses [9, 10]. Several cells respond to mechanical forces by activating MAPK pathways [11]. Mechanical stress has been shown to stimulate ERK1/2 in pulmonary epithelial cells [12] and endothelial cells [13]. ERK1/2 activation upon cyclic stretch has been reported in human bronchial epithelial cells [4] and primary rat alveolar epithelial cells [14]. In an *in vitro* model of VILI, cyclic stretch-induced interleukin-8 production from lung epithelial cells was reduced by ERK1/2 inhibition [4]. *In vivo*, bleomycin plus ventilation-induced lung fibrosis was attenuated in mice with pharmacologic inhibition of ERK1/2 [15].

Given the important role of ERK1/2 in cell proliferation, ERK1/2 inhibitors have been investigated as candidate targets in cancer and rheumatoid arthritis [16–18]. Nevertheless, defective ERK1/2 signaling has been associated with autoimmunity [19] raising concerns for the use of ERK1/2 inhibition in inflammatory diseases, emphasizing the need for more selective therapeutic targets [18].

Activation of each of the three MAPKs is controlled by several MAP3Ks ensuring the specificity of signaling responses. Tumor progression locus 2 (Tpl2) is a MAP3kinase that phosphorylates and activates the extracellular signal-regulated kinase (ERK1/2) [20–22]. Tpl2 was originally identified as a proto-oncogene, but is now recognized to play an important role in regulating ERK1/2 signaling in multiple cell types, including T-cells, macrophages, and epithelial cells [22–26]. *In vitro* studies using Tpl2 overexpressing cells have shown that Tpl2 activates ERK, JNK, p38, and the transcription factors NFAT (nuclear factor of activated T cells) and NF-κB [23, 27]. *In vivo*, expression of a constitutively active form of Tpl2 under the control of a T cell-specific promoter in mice resulted in development of thymic lymphomas [21]. On the contrary, Tpl2-deficent (Tpl2^-/-^) mice remain healthy throughout their normal life span [28]. Studies on Tpl2^-/-^mice have proven that Tpl2 participates in signal transduction of TLR, T-cell receptors, G protein-coupled receptors, tumor necrosis factor (TNF), and CD-40 [25, 28–30]. In these studies, the anti-inflammatory effects of Tpl2 ablation were recognized. Tpl2^-/-^macrophages produce less inflammatory mediators upon lipopolysacharite (LPS) stimulation [29], and Tpl2^-/-^mice are resistant to LPS-induced shock [28]. In a model of acute pancreatitis, lung inflammation was less in Tpl2^-/-^mice [31], and in a model of experimental colitis, Tpl2^-/-^mice and mice treated with a Tpl2 inhibitor had less bowel inflammation [32].

The present study examined the hypothesis that genetic and pharmacologic inhibition of Tpl2 can ameliorate VILI. We first compared naïve wild type (WT) and Tpl2^-/-^mice, as well as WT and Tpl2^-/-^mice ventilated for 4 h with normal tidal volume (VT), to exclude any unexpected effect of Tpl2 deficiency on lung mechanics, response to anesthesia, or normal-VT ventilation. We then subjected WT and Tpl2^-/-^mice to mechanical ventilation with high VT, under conditions previously shown to induce severe VILI in WT mice [33]. Finally, we examined the effect of pharmacologic inhibition of Tpl2 in WT mice subjected to mechanical ventilation with high VT, when given as a pretreatment, before the initiation of high VT, and, in a more clinically relevant approach, given after initiation of high VT ventilation.

## Methods

### Animal experiments

**Figure 1:**
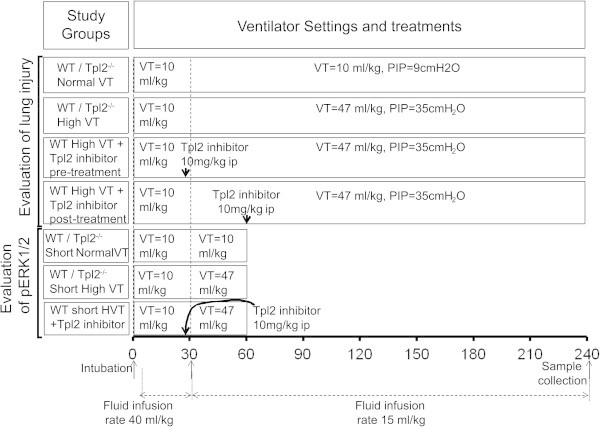
**Experimental procedure.** At time 0, animals were anesthetized, tracheostomized, and connected to the ventilator. Through the carotid artery, fluid and anesthesia were administered at a rate of 40 ml/kg/h for 30 min after the initiation of ventilation. At 30 min, fluid infusion rate changed to 15 ml/kg/h in all groups, and ventilator settings were changed as indicated in groups allocated to high VT ventilation. The Tpl2 inhibitor-treated groups received an intraperitoneal injection of Tpl2 inhibitor (thick black arrow), either 5 min prior to the initiation of high VT ventilation (pretreatment) or 30 min after high VT ventilation (posttreatment).

We studied 12 groups of mice, a total of 58 male C57BL6 (WT) and 44 Tpl2^-/-^mice on C57Bl6 background [28] at 8 to 10 weeks of age (25- to 30-g weight). The experimental groups and number of mice in each group are presented in Table 1, and the experimental procedure is shown in Figure 1. Mice were obtained from the Foundation of Research and Technology Institute Animal Facility. All experiments were approved by the Research Animal Care Committee of University of Crete Medical School and Heraklion Prefecture Veterinary Authority. All experiments were performed at the experimental Intensive Care Medicine laboratory at the Medical School of the University of Crete.

**Table 1:** **Experimental groups**

Strain treatment	Sample ***(N)***	
	WT	Tpl2 ^-/-^
Control-PV curve and sample collection	10	10
Normal VT ventilation	8	8
High VT ventilation	8	8
High VT ventilation + Tpl2 inhibitor pretreatment	5	
High VT ventilation + Tpl2 inhibitor posttreatment	5	
Control-no ventilation (ERK1/2 phosphorylation)	6	6
Normal VT 60 min (ERK1/2 phosphorylation)	4	4
Normal VT 30 min + High VT 30 min (ERK1/2 phosphorylation)	8	8
Normal VT 30 min + High VT 30 min (ERK1/2 phosphorylation) + Tpl2 inhibitor pretreatment	4

The effects of mechanical ventilation on the development of lung injury were studied in WT and Tpl2^-/-^mice ventilated with either normal or high tidal volume (VT), as detailed below, and described previously [33]. Mice were anesthetized with intraperitoneal (i.p.) injection of ketamine 100 mcg/g and fentanyl 0.12 mcg/g [33], tracheostomized, and ventilated using SAP830 ventilator (IITC Life Science, Woodland Hills, CA, USA). Ventilation settings for normal VT were VT = 10 ml/kg resulting in peak inspiratory pressure (PIP) = 9 ± 0.5 cmH_2_O, positive end-expiratory pressure PEEP =1.5 cmH_2_O, respiratory rate (RR) =130 breaths/min, with recruitment maneuvers performed every 30 min. For high VT, ventilation settings were changed from normal VT after 30 min of hemodynamic stabilization, to high VT = 47 ± 2 ml/kg, targeted to a PIP = 35 ± 0.5 cmH_2_O, RR = 60 breaths/min, PEEP = 1.5 cmH_2_O, without recruitment maneuvers. Fraction of inspired oxygen (FiO_2_) was 30% in all experiments, and inhaled carbon dioxide (CO_2_) was added in high VT groups to prevent hypocapnia. Arterial blood pressure, PIP, PEEP, and VT were monitored throughout the study. At the end of the 240-min experiment, blood was collected from the arterial line for blood gas analysis, followed by inspiratory pressure volume curve, bronchoalveolar lavage fluid (BALF) and tissue collection. For histological evaluation, lungs from mice not subjected to BALF collection were inflated with 4% paraformaldehyde at a transpulmonary pressure of 25 cmH_2_O.

The possible protective effects of pharmacologic Tpl2 inhibition were studied in WT mice ventilated with high VT as described above, and treated with a Tpl2 inhibitor (Calbiochem #616404, USA) 10 mg/kg, 20-μl DMSO in a 0.2-ml normal saline given as a single i.p. injection as per manufacturer's instructions, and previous reports [34]. Two groups of WT mice treated with Tpl2 inhibitor were studied: the pretreatment group, in which the inhibitor was given 5 min prior to the initiation of high VT ventilation, and the posttreatment group, in which the inhibitor was given 30 min after high VT ventilation.

As controls served WT and Tpl2^-/-^mice ventilated briefly (approximately 1 min), until paralyzed, to obtain an inspiratory pressure volume curve and subsequently BALF and tissue samples.

The activation of ERK1/2 induced by mechanical ventilation, which is known to occur even after very brief periods of ventilation [14, 15] was studied in WT and Tpl2^-/-^mice in the same study groups, but total ventilation time was limited to 60 min. Due to the rapid activation of ERK1/2 upon mechanical ventilation different controls were required for this experiment. Specifically, as controls served mice of both genotypes not subjected to mechanical ventilation, euthanized with pentobarbital. In these experiments, only BALF cells and lungs were collected.

### Evaluation of lung injury

Ventilator-induced lung injury is characterized by high-permeability pulmonary edema and inflammation. The presence of high-permeability pulmonary edema was evaluated using lung compliance, oxygenation, and BALF protein concentration. The presence of inflammation was evaluated by BALF cytokines. Lung histological evaluation also provided information on alveolar membrane integrity and presence of inflammatory cellular infiltration. As an indicator of lung compliance, we used the inspiratory capacity, defined as the volume to inflate the lungs to an airway pressure of 25 cmH_2_O [33]. Results are expressed as percentage (%) of control and normalized to body weight due to the differences in weight of study mice. Concentration of proteins in BALF was measured using bicinchoninic acid assay (Pierce Chemical Co, Rockford, IL, USA). The levels of the proinflammatory cytokines, interleukin-6 (IL-6), and macrophage inflammatory protein 2 (MIP-2), were measured in BALF ELISAs (R&D Systems Inc, Minneapolis, MN, USA). Paraffin-embedded lung sections, sectioned 6-μm thick and stained with hematoxylin and eosin, were analyzed by a pathologist blinded to the treatment groups. In each group, 20 random high power fields (×400) were scored, for five independent variables: neutrophils in alveolar spaces, neutrophils in interstitial spaces, hyaline membranes, proteinaceous debris filling the airspaces, and alveolar septal thickening, as previously described [35]. The resulting injury score is a continuous value between 0 and 1.

### Evaluation of ERK1/2 phosphorylation in WT and TPL2^-/-^mice after high VT ventilation

Levels of ERK1/2 phosphorylation were evaluated in BALF cells by flow cytometry and in lung homogenates by Western blot. BALF cells were immediately fixed in 1.5% formaldehyde permeabilized by ice-cold methanol for 10 min and then washed and re-suspended in PBS (Ca^2+^- and Mg^2+^-free) containing 0.1 mM EDTA, 5% FBS, and 0.05% NaN_3_. BALF cells were first incubated with rabbit anti-mouse phospho-p44/42 MAPK (ERK1/2) (Thr202/Tyr204) antibody (Cell Signaling Techn, Danvers, MA, USA) for 1 h at 4°C. Then, cells were incubated for 20 min at 4°C with FITC goat anti-rabbit IgG (BD Biosciences, Franklin Lakes, NJ, USA). To discriminate alveolar macrophages, cell surface staining was carried out by incubation with PerCP-Cy5.5 anti-mouse CD11c (Biolegent, San Diego, CA, USA) for 30 min at 4°C. Appropriate isotype control was also used. The flow cytometry events were acquired in a MoFlo Legacy Cell Sorter (Beckman Coulter, Inc., Fullerton, CA, USA) and analyzed with the use of Summit Software (Summit Software, Inc., Fort Wayne, IN, USA). For Western blot analysis, a 100-μl tissue sample was suspended in 500 μl of lysis buffer containing 50 mM Tris (pH 6.8), 2% sodium dodecyl sulfate, 5 mM EDTA, and protease inhibitors (Complete, Boehringer, Ingelheim, Germany), homogenized, ultrasonicated, and centrifuged. A 0.1* volume of loading buffer (containing 0.3% bromophenol blue, 50% glycerol, 0.3% mercaptoethanol, and 50% [*v*/*v*] lysis buffer) was added. Samples were separated by sodium dodecyl sulfate-polyacrylamide gel electrophoresis and transferred to nitrocellulose membranes. Membranes were labeled with a phospho- and total ERK1/2 primary antibody (Cell Signaling Technology, Danvers, MA, USA). Washed membranes were incubated with goat anti-rabbit anti-serum conjugated with horseradish peroxidase (Amersham International, Amersham, UK). Antigen-antibody complexes on the membranes were detected by enhanced chemiluminescence (Amersham).

### Statistical analysis

Data were compared by one-way ANOVA, using the Shapiro-Wilk normality test, and the Kruskal-Wallis test for non-parametric data with Dunn's multiple comparisons posttest, with SigmaStat software. For each of the parameter evaluated, the comparisons made included: comparison between similarly ventilated animals of different genotype or treatment, and of ventilated animals with their genotype-matched controls. All data in text are expressed as means ± SD. Significance was defined as *p* < 0.05.

## Results

### Anesthesia and mechanical ventilation for 240 min were tolerated by WT and TPL2^-/-^mice

Blood pressure averaged 92 ± 20 mmHg after 30 min of MV and 93 ± 23 mmHg at the end of the experiment in WT mice, and 98 ± 21 and 105 ± 24 mmHg, respectively, in Tpl2^-/-^mice (*p* > 0.05 for all comparisons). All mice survived the 240 min of mechanical ventilation.

### WT and TPL2^-/-^mice do not differ at baseline and after mechanical ventilation with normal VT

**Figure 2:**
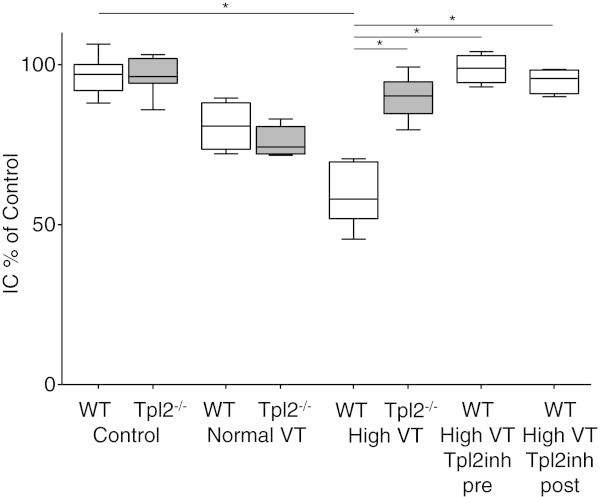
**Lung mechanics.** Inspiratory capacity, defined as the volume (in ml/kg) to inflate the lungs to an airway pressure of 25 cmH_2_O, expressed as percentage (%) of control, of wild-type (WT, white boxes), and Tpl2-deficient mice (Tpl2^-/-^, gray boxes) subjected only to sample collection (control, *n* = 8 per group), mechanical ventilation with normal tidal volume (VT) for 240 min (*n* = 7 to 9 per group), or high VT for 210 min (*n* = 8 to 9 per group), and WT mice subjected to 210 min of high VT ventilation treated with a Tpl2 inhibitor, either prior (Tpl2inh pre, *n* = 5) or after initiation of high VT ventilation (Tpl2inh post, *n* = 5). Wild-type mice subjected to high VT ventilation had lower inspiratory capacity compared to control WT mice, as well as compared to similarly ventilated Tpl2^-/-^mice, and WT mice treated with the Tpl2 inhibitor, *p < 0.01. Data are presented in box plots, where boxes represent 25th to 75th percentile; line represent median and whisker represent min and max, in all figures.

**Figure 3:**
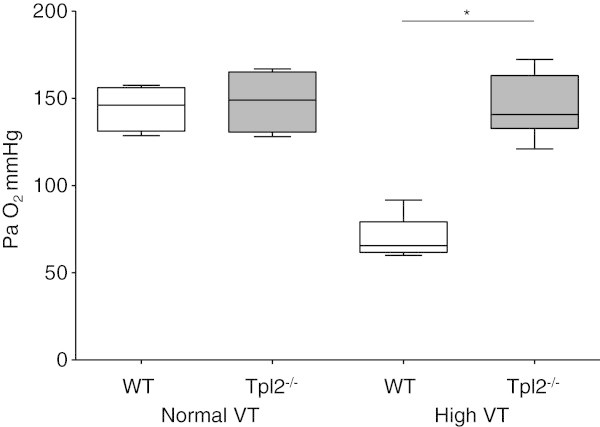
**Oxygenation.** Arterial blood gas PaO_2_ from wild-type (WT, white boxes), and Tpl2-deficient mice (Tpl2^-/-^, gray boxes) subjected to mechanical ventilation with normal tidal volume (VT) for 240 min, or high VT for 210 min (*n* = 6 to 8 per group). PaO_2_ was lower in WT mice subjected to high VT than in similarly ventilated Tpl2^-/-^mice, *p < 0.05.

**Figure 4:**
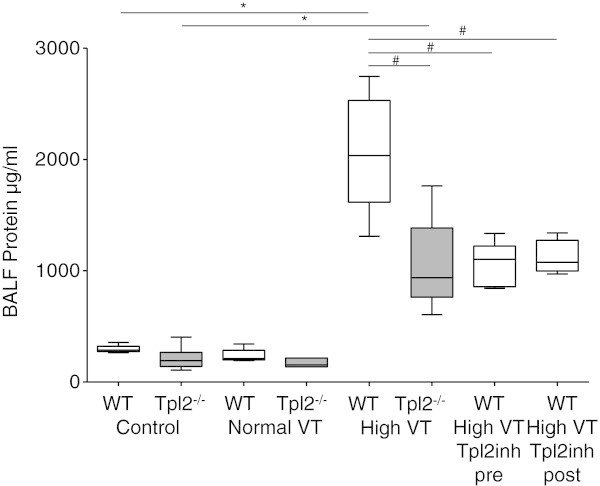
**BALF protein.** Bronchoalveolar lavage fluid (BALF) concentration of protein from wild-type (WT, white boxes), and Tpl2-deficient mice (Tpl2^-/-^, gray boxes) subjected only to sample collection (control, *n* = 7 to 8 per group), mechanical ventilation with normal tidal volume (VT) for 240 min (*n* = 6 to 8 per group), or high VT for 210 min (*n* = 7 to 9 per group), and WT mice subjected to 210 min of high VT ventilation treated with a Tpl2 inhibitor, either prior (Tpl2inh pre, *n* = 5) or after initiation of high VT ventilation (Tpl2inh post, *n* = 5). BALF proteins' concentration was higher in WT and Tpl2^-/-^mice subjected to high VT ventilation, than in control WT and Tpl2^-/-^mice, respectively, *p < 0.001. BALF proteins' concentration was higher in WT mice subjected to high VT ventilation than in similarly ventilated Tpl2^-/-^mice, and WT mice treated with the Tpl2 inhibitor, ^#^ *p* < 0.01.

**Figure 5:**
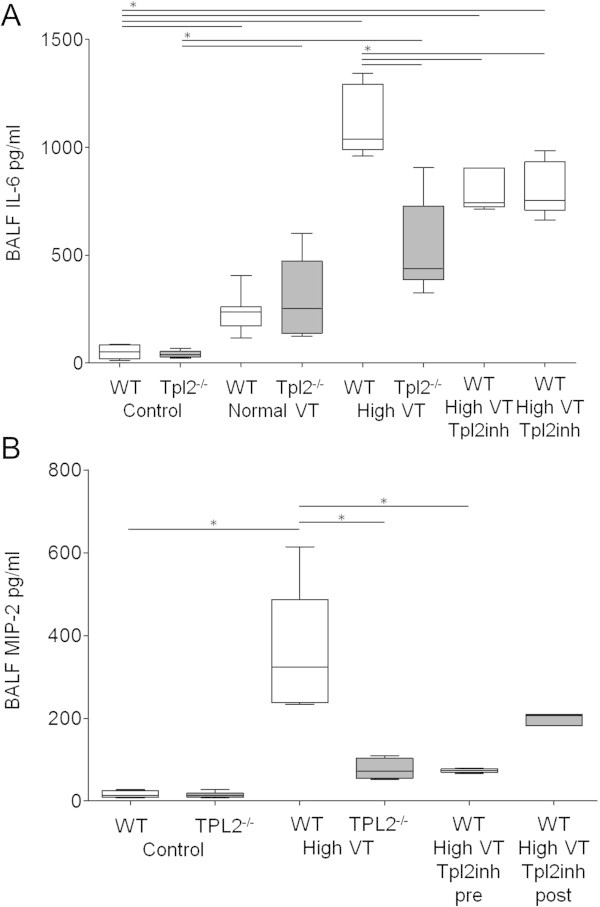
**Bronchoalveolar lavage fluid (BALF) concentration of (A) IL-6 and (B) MIP-2, from wild-type (WT, white boxes), and Tpl2-deficient mice (Tpl2** ^**-/-**^ **, gray boxes) subjected only to sample collection (control, n=5-8), mechanical ventilation with normal tidal volume for 240 min, or high tidal volume for 210 min (n=5-7), and WT mice subjected to 210 min of high tidal volume ventilation treated with a Tpl2 inhibitor, either prior (Tpl2inh pre, n=5), or after initiation of high VT ventilation (Tpl2inh post, n=5).** BALF IL-6 was lower in genotype-matched control mice than in ventilated mice, *p<0.05. High tidal volume-induced increase in BALF IL-6 was greater in WT untreated mice, than in Tpl2^-/-^ mice, and WT mice treated with the Tpl2 inhibitor, *p<0.05. BALF MIP-2 concentration was higher in WT mice ventilated with high VT than in WT control mice *p<0.001. BALF MIP-2 concentration was also higher in WT mice ventilated with high VT than in similarly ventilated Tpl2^-/-^ mice, and WT mice pre-treated with the Tpl2 inhibitor, *p<0.001.

Control WT and Tpl2^-/-^mice had no differences on histological appearance, lung compliance, and concentrations of proteins, IL-6 and MIP-2 in BALF. Similarly, after 240 min of mechanical ventilation with normal VT, WT, and Tpl2^-/-^, the mice had no differences in all indices of lung injury studied, inspiratory capacity (Figure 2), arterial oxygen (Figure 3), and concentration of proteins (Figure 4) and IL-6 (Figure 5) in BALF. Mechanical ventilation with normal VT resulted in a similar increase in BALF IL-6 concentration in both strains.

### Genetic deficiency of Tpl2^-/-^protects from high VT ventilation-induced lung injury

Mechanical ventilation with high VT-induced high-permeability pulmonary edema that was more severe in WT than in Tpl2^-/-^mice. After 210 min of high VT ventilation, lung compliance decreased in WT mice to 60% of control, but not in Tpl2^-/-^mice (93% of control; Figure 2). The development of pulmonary edema was associated with impaired oxygenation (Figure 3). Tpl2^-/-^mice subjected to high VT ventilation had higher PaO_2_ than WT mice (145 ± 18 vs. 70 ± 13 mmHg, *p* < 0.05, Figure 2). There were no differences in PaCO_2_, pH, and lactate between the two genotypes after high VT ventilation (data not shown). The concentration of proteins in BALF increased after high VT ventilation from baseline in WT and Tpl2^-/-^mice (*p* < 0.001 vs. corresponding controls). Yet, BALF proteins concentration was lower in Tpl2^-/-^than in WT mice after high VT ventilation (1,047 ± 385 vs. 2,051 ± 495 μg/ml, *p* < 0.01, Figure 4).

**Figure 6:**
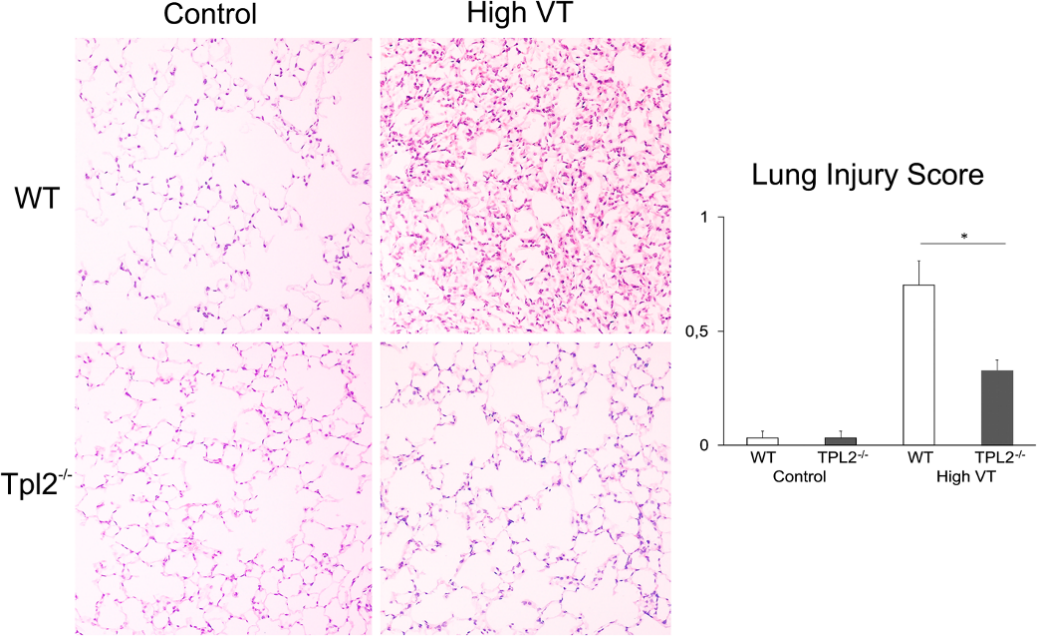
**Histology.** Representative photographs of hematoxylin-eosin-stained lung sections, ×200 magnification, from control wild-type and Tpl2-deficent mice and from wild-type and Tpl2-deficient mice subjected to high tidal volume ventilation for 210 min (*n* = 4 per group) and calculated lung injury score. Mechanical ventilation with high VT-induced lung injury that was more severe in WT than in Tpl2^-/-^mice *p < 0.01.

Mechanical ventilation with high VT induced an inflammatory response in the lungs of mice of both genotypes, but more severe in WT than in Tpl2^-/-^mice. BALF concentration of IL-6 increased from control, more in WT than in Tpl2^-/-^mice, while the concentration of MIP-2 in BALF increased only in WT mice and not in Tpl2^-/-^mice (Figure 5A,B). In the histological examination, lungs from WT mice subjected to high VT ventilation presented severe injury with the presence of mixed inflammatory infiltrates (neutrophils and alveolar macrophages) in the interstitial and alveolar spaces, edema, and thickening of the alveolar walls, and a lung injury score of 0.7. Lungs from Tpl2^-/-^mice subjected to high VT ventilation presented only sparse, very mild inflammatory infiltrates, and a lung injury score of 0.33 (Figure 6).

### Pharmacologic inhibition of Tpl2 protects from high VT ventilation-induced lung injury

The observation that Tpl2 deficiency is protective in our VILI model prompted us to examine the potential therapeutic effect of pharmacologic inhibition of Tpl2 in high VT-induced lung injury. First, we examined if pretreatment with Tpl2 inhibitor would ameliorate VILI in WT mice. When WT mice were subjected to high VT ventilation, no differences were observed between untreated mice and mice receiving an i.p. injection of DMSO in normal saline (vehicle) in any of the parameters evaluated (data not shown), and therefore, untreated mice were used as controls. Pretreatment with the Tpl2 inhibitor was effective in preventing high VT-induced decrease in lung compliance (Figure 2). Additionally, high VT-induced increase in the concentrations of protein, IL-6 and MIP-2, observed in ventilated, untreated WT mice, was less in mice pretreated with the Tpl2 inhibitor (Figures 4 and 5).

Although pretreatment was effective, such therapeutic approach is rarely feasible in clinical practice. We, therefore, tested the effects of pharmacological Tpl2 inhibition when the inhibitor was administered 30 min after the initiation of high VT ventilation. We found that posttreatment with the Tpl2 inhibitor was also effective in ameliorating indices of VILI. Lung compliance and concentrations of protein and IL-6 in BALF were similar between WT subjected to high VT ventilation and treated with the Tpl2 inhibitor either before or after initiation of high VT, and always lower than in untreated mice (Figures 2, 3, 4, and 5). Although BALF concentration of MIP-2 was similar in mice treated with the Tpl2 inhibitor before or after high VT, only the pretreated group had significantly lower MIP-2 than the untreated group (Figure 5B).

### High VT ventilation-induced ERK1/2 phosphorylation is decreased in alveolar macrophages of Tpl2^-/-^mice and WT mice treated with the Tpl2 inhibitor

**Figure 7:**
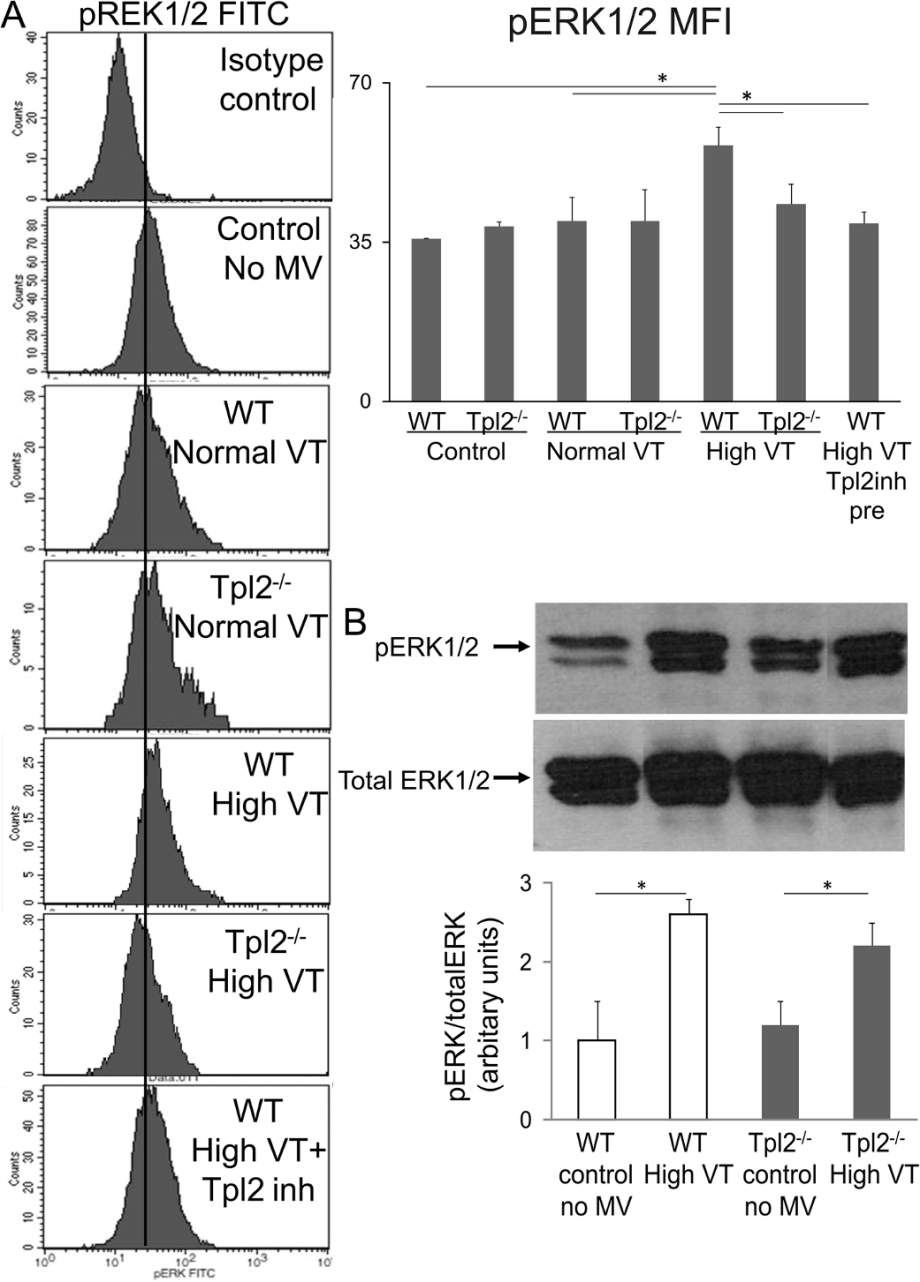
**ERK1/2 phosphorylation. (A)** The histograms are representative of p-ERK1/2 fluorescence intensity of BALF CD11c positive cells (alveolar macrophages) from non-ventilated mice (*n* = 6), and from wild-type and Tpl2-deficient mice exposed to either 60 min of normal tidal volume ventilation (*n* = 4 per group) or 30 min of normal tidal volume ventilation followed by 30 min of high tidal volume ventilation (*n* = 4 per group), with or without treatment with a Tpl2 inhibitor given 5 min prior to high tidal ventilation. BALF cells stained with isotype control are also depicted. ERK1/2 phosphorylation, as indicated by mean fluorescent intensity (MFI), was higher in WT mice subjected to high VT ventilation than in WT control mice and WT mice ventilated with normal VT *p < 0.05. ERK1/2 phosphorylation was also higher in WT mice subjected to high VT ventilation than in similarly ventilated Tpl2^-/-^mice and WT mice treated with the Tpl2 inhibitor *p < 0.05. **(B)** Representative Western blot analysis for phosphorylated and total ERK1/2 of lung homogenates from non-ventilated mice (*n* = 4 per group) and from WT and Tpl2^-/-^mice (*n* = 4 per group) exposed to 30 min of high tidal volume ventilation. Mechanical ventilation was associated with increased ERK1/2 phosphorylation in lungs of both WT and Tpl2^-/-^mice compared to genotype-matched control mice, *p < 0.05.

It is known that ERK1/2 activation contributes to the inflammatory response induced by high VT ventilation, and that Tpl2 is an essential regulator of ERK1/2 activation. We therefore examined ventilation-induced activation of ERK1/2 in alveolar macrophages and total lungs homogenates from WT and Tpl2^-/-^mice. BALF cells from control, non-ventilated mice and from mice subjected to 60 min of mechanical ventilation consists of >85% alveolar macrophages (data not shown). Mechanical ventilation with high VT but not normal VT increased ERK1/2 phoshorylation in alveolar macrophages of WT mice (Figure 7). This increase in levels of phospho-ERK1/2 after high VT ventilation was not observed in alveolar macrophages from Tpl2^-/-^mice and in those from WT mice treated with the Tpl2 inhibitor. Levels of phospho-ERK1/2 were similar in lung homogenates from WT and Tpl2^-/-^mice ventilated on high VT.

## Discussion

The inflammatory response and barrier dysfunction that characterize acute lung injury can be exacerbated by mechanical ventilation, and, apart from low tidal volume ventilation, no effective treatment is available yet. Identifying the intracellular signaling molecules involved in VILI is thus important for designing novel therapeutic approaches. In the present study we show that inhibition of Tpl2, a MAP3K kinase, is protective in a mouse model of VILI. Indices of high-permeability pulmonary edema and lung inflammation induced by injurious ventilation were lower in Tpl2-deficient mice than in WT ones. More importantly, this protective effect was reproduced using pharmacologic inhibition of Tpl2 in WT mice.

It is well established that injurious ventilation induces high-permeability pulmonary edema and inflammation. Studies in ARDS patients have shown that even brief periods of injurious ventilation increase BALF inflammatory mediators [36]. In mouse models, high tidal volume ventilation induces lung injury characterized by pulmonary edema with increased concentrations of proteins and cytokines in BALF and inflammatory cell infiltration and diffuse alveolar damage on histology, a picture similar to human ARDS [1]. In this study, high VT ventilation-induced lung injury in WT mice, characterized by deterioration in lung mechanics and oxygenation, increased concentration of proteins and cytokines in BALF, and inflammatory cell infiltration on histology. Tpl2 deficiency was associated with less lung injury upon high VT ventilation. Specifically, Tpl2-deficient mice showed no deterioration of lung mechanics and oxygenation, less injury on histology, and lower levels of BALF proteins and cytokines than WT mice, suggesting a protective role of Tpl2 inhibition in VILI.

The ERK1/2 pathway is a highly conserved signaling pathway involved in fundamental cellular processes such as growth, differentiation, and survival. ERK1/2 is activated by a wide variety of receptors including G protein-coupled receptors (GPCR), tyrosine kinase receptors, TLRs, ion channels, and others [9, 10]. Studies have shown that ERK1/2 is also involved in mechanotransduction [1], and that ERK1/2 activation by mechanical stretch contributes to the inflammatory response induced by injurious ventilation [4, 15].

Tpl2 is an essential regulator of ERK1/2 activation, mediating signals initiated by cytokine or Toll-like receptors to induce pro-inflammatory cytokine production [22, 28]. In disease models, Tpl2^-/-^mice were initially found to be resistant to LPS/d-galactosamine-induced endotoxin shock [28]. Recent studies showed that Tpl2 deficiency was associated with reduced adipose tissue inflammation in diet-induced obesity, and reduced acetaminophen-induced liver injury [37, 38]. In an experimental colitis model, pharmacologic as well as genetic inhibition of Tpl2 was found effective in reducing bowel inflammation [32]. Yet, the role of Tpl2 in inflammatory lung diseases has not been investigated. Involvement of Tpl2 in lung inflammation has been only indirectly demonstrated, since Tpl2 was found up-regulated in a proteome analysis of lung tissues from rats exposed to cigarette smoke [39], and mice lacking Tpl2 had reduced lung inflammation in a model of acute pancreatitis [31]. This study showed that Tpl2 is involved in barrier dysfunction and inflammation triggered by injurious ventilation, as inhibition of Tpl2 ameliorated lung inflammation and high-permeability pulmonary edema.

The pathway consistently shown to be impaired in the absence or inhibition of Tpl2 is the ERK1/2 pathway [25, 30–32, 37]. Also in this study, high tidal volume-induced ERK1/2 activation was lower in alveolar macrophages from Tpl2^-/-^mice and from WT mice treated with the Tpl2 inhibitor than from WT untreated mice. The observation that ERK1/2 activation was similar in lung homogenates of WT and Tpl2^-/-^mice could be explained by the presence of other ERK1/2 activating signals, not mediated by Tpl2, such as TGFβ or other growth factors. The possible inhibition of other kinases was not examined and cannot be ruled out.

An important finding of this study is that pharmacologic inhibition of Tpl2, and not only genetic, was effective in ameliorating VILI. Systemic administration of a Tpl2 inhibitor protected from VILI, both when given as pretreatment and when given after initiation of injurious ventilation. A time point well after establishing high VT (30 min) was chosen as posttreatment, reasoning that ERK1/2 activation occurs sooner, even within 5 min after high stretch [14, 15], and that a deterioration of lung injury resulting in high airway pressures would be recognized by a clinician within 30 min and prompt therapeutic interventions. WT mice receiving the Tpl2 inhibitor after being on high VT ventilation for 30 min had similar lung mechanics, and BALF protein, IL-6 and MIP-2 concentrations as Tpl2^-/-^mice at the end of the experiment, and lower than untreated, ventilated on high VT, WT mice. The only observed difference was that the delayed treatment with the Tpl2 inhibitor was not able to prevent the increase in BALF concentration of MIP2 induced by injurious ventilation. These findings suggest that the protective effect of Tpl2 inhibition is quite well maintained when the inhibitor is administered after an increase in airway pressure is observed, as it would happen in clinical practice.

The potential advantage of Tpl2 as therapeutic target over ERK1/2 is its selective activation by inflammatory stimuli with a consequent reduction in side effects. Thus, inhibition of Tpl2 will not affect activation of ERK1/2 by other agonists, like growth factors, which has been proven protective in several diseases, such as myocardial and cerebral ischemia-reperfusion injury [40, 41]. Small molecule inhibitors designed to suppress Tpl2 were able to inhibit pro-inflammatory cytokine production from LPS-treated human primary macrophages [42], supporting the therapeutic potential of Tpl2 inhibition. Additionally, inhibition of Tpl2 has been a promising target for inflammatory diseases including inflammatory bowel disease, rheumatoid arthritis and liver disease [42–44], and Tpl2 inhibitors are among the ones to be tested in clinical trials [18].

Of course the potential adverse effects of Tpl2 inhibition have been evaluated neither in clinical practice nor under experimental conditions. Although Tpl2^-/-^mice appear to be prone to chemically induced carcinogenesis in several animal models [22], they do not develop spontaneous cancers, and the clinical importance of Tpl2-deficiency-mediated carcinogenesis in acute inflammatory conditions, such as VILI is probably small; as such treatments would be given for only brief periods of time. Data on toxicity of Tpl2 inhibitors are lacking, but an inhibitor of MEK1/2 (mitogen-activated protein kinase kinase), a kinase that is upstream of ERK1/2 in the Raf-MEK-ERK pathway, has been investigated in phase I and phase II clinical trials in patients with advanced malignancies and proved to have a good safety profile [45–48]. A more important concern of Tpl2 inhibition is the potential immune suppression. Indeed, Tpl2 ablation ameliorated macrophages response to LPS [28, 49], increased susceptibility of mice to *Listeria monocytogenes* infection [50], and decreased clearance of *Toxoplasma gondii* [51]. It is possible that Tpl2 inhibition could adversely affect critically ill patients with ongoing infection. This is a common limitation of all anti-inflammatory therapeutic interventions, such as corticosteroids in ALI-VILI.

In this study, we use our established model of aseptic, ‘one-hit’ VILI [33], to clarify the role of Tpl2. This ‘one-hit’ model is widely used in studies to investigate the pathogenesis of VILI. Using this model, we were able to confirm that inhibition of Tpl2 ameliorates indices of high-permeability pulmonary edema and inflammation induced by injurious ventilation. Nonetheless, this ‘one-hit’ model has inherent limitations. Specifically, a very high, not clinically relevant tidal volume has to be used. We and others have shown [33, 52, 53] that only such high volume can result in distortion of lung units required to induce VILI in healthy mice. Yet, it is now clear that in patients with ARDS some alveoli are exposed to such high distending pressures [54]. Additionally, to compensate for the hemodynamic effects of high intrathoracic pressures, mice receive fluid loading, that may contribute to the development of pulmonary edema. As all groups of ventilated mice received the same amount of fluids, but only the group of WT mice ventilated on high tidal volume developed significant edema, it is reasonable to assume that fluid loading may only exacerbate the high tidal volume-induced alteration in alveolar permeability.

## Conclusion

In conclusion, Tpl2 contributes to the pathogenesis of high-permeability pulmonary edema and inflammation induced by high tidal volume ventilation, as genetic deficiency of Tpl2 appears to be protective in this model of murine VILI. Additionally, pharmacologic inhibition of Tpl2 is effective in ameliorating indices of VILI, even when given after establishing injurious ventilation, suggesting a potential therapeutic role for Tpl2 inhibitors in VILI.

## Authors’ information

E Kaniaris, MD, is a fellow in pulmonary medicine and graduate student at the Department of Intensive Care Medicine, Experimental Intensive Care Medicine Laboratory, KV, MD, PhD, is an assistant professor in Intensive Care Medicine, PI at the Experimental Intensive Care Medicine Laboratory, EV, MD, is a fellow in pediatrics and graduate student at the department of Clinical Chemistry, ET, MD, is a graduate student at the Department of Intensive Care Medicine. E Kondili, MD, PhD, is an assistant professor in the Department of Intensive Care Medicine; EL, MD, is a pathologist at the Department of Pathology. CT, PhD, is associate professor in Clinical Chemistry, head of the Laboratory of Clinical Chemistry, and member of the research team who generated the Tpl2-deficient mice, and DG, MD, PhD, is a professor and head of the Department of Intensive Care Medicine. All departments and authors are affiliated to the Medical School of the University of Crete in Greece.

## Abbreviations

ALI: acute lung injury
BALF: bronchoalveolar lavage
ERK1/2: extracellular signal-regulated kinase 1/2
GPCR: G protein-coupled receptors
IL-6: interleukin-6
JNK: C-Jun N-terminal kinase
MAPK: mitogen-activated serine/threonine kinase
MEK1/2 (or MAP2K): mitogen-activated protein kinase kinase
MAP3K: mitogen-activated protein kinase kinase kinase
MIP-2: macrophage inflammatory protein 2
PIP: peak inspiratory pressure
Tpl2: tumor progression locus 2
Tpl2-/-: Tpl2 deficient
VILI: ventilator-induced lung injury
VT: tidal volume
WT: wild-type.
